# Distress related to myocardial infarction and cardiovascular outcome: a retrospective observational study

**DOI:** 10.1186/1471-244X-11-98

**Published:** 2011-06-10

**Authors:** Roland von Känel, Roman Hari, Jean-Paul Schmid, Hugo Saner, Stefan Begré

**Affiliations:** 1 Department of General Internal Medicine, Division of Psychosomatic Medicine, Inselspital, Bern University Hospital, and University of Bern, Switzerland; 2 Swiss Cardiovascular Center, Cardiovascular Prevention and Rehabilitation, Inselspital, Bern University Hospital, and University of Bern, Switzerland

**Keywords:** Myocardial infarction, pain, retrospective study, psychological stress, risk factor

## Abstract

**Background:**

During acute coronary syndromes patients perceive intense distress. We hypothesized that retrospective ratings of patients' MI-related fear of dying, helplessness, or pain, all assessed within the first year post-MI, are associated with poor cardiovascular outcome.

**Methods:**

We studied 304 patients (61 ± 11 years, 85% men) who after a median of 52 days (range 12-365 days) after index MI retrospectively rated the level of distress in the form of fear of dying, helplessness, or pain they had perceived at the time of MI on a numeric scale ranging from 0 ("no distress") to 10 ("extreme distress"). Non-fatal hospital readmissions due to cardiovascular disease (CVD) related events (i.e., recurrent MI, elective and non-elective stent implantation, bypass surgery, pacemaker implantation, cerebrovascular incidents) were assessed at follow-up. The relative CVD event risk was computed for a (clinically meaningful) 2-point increase of distress using Cox proportional hazard models.

**Results:**

During a median follow-up of 32 months (range 16-45), 45 patients (14.8%) experienced a CVD-related event requiring hospital readmission. Greater fear of dying (HR 1.21, 95% CI 1.03-1.43), helplessness (HR 1.22, 95% CI 1.04-1.44), or pain (HR 1.27, 95% CI 1.02-1.58) were significantly associated with an increased CVD risk without adjustment for covariates. A similarly increased relative risk emerged in patients with an unscheduled CVD-related hospital readmission, i.e., when excluding patients with elective stenting (fear of dying: HR 1.26, 95% CI 1.05-1.51; helplessness: 1.26, 95% CI 1.05-1.52; pain: HR 1.30, 95% CI 1.01-1.66). In the fully-adjusted models controlling for age, the number of diseased coronary vessels, hypertension, and smoking, HRs were 1.24 (95% CI 1.04-1.46) for fear of dying, 1.26 (95% CI 1.06-1.50) for helplessness, and 1.26 (95% CI 1.01-1.57) for pain.

**Conclusions:**

Retrospectively perceived MI-related distress in the form of fear of dying, helplessness, or pain was associated with non-fatal cardiovascular outcome independent of other important prognostic factors.

## Background

Myocardial infarction (MI) is an unexpected life-threatening event which is perceived as stressful by many patients who may expect death or serious disability [1,2]. For instance, after symptom onset three out of four patients with an acute coronary syndrome (ACS) indicated to have experienced moderate or high levels of MI-related distress, including being frightened and thinking they might be dying when symptoms came on [3]. In another study, fear of dying and perceived severity of MI (e.g. fright of recurrent chest pain) together accounted for more than half of the variance in distress perceived during MI [4]. Fear of dying and distress were also highly associated with intensity of chest pain at the time of MI [3]. Given that chest pain experience is greatly modulated by affective states [5], chest pain intensity was discussed as an equivalent of emotional distress perceived at the time of MI [3].

Distress during ACS profoundly impacts psychological adjustment in the wake of the cardiac event, particularly bringing on symptoms of anxiety, depression, and posttraumatic stress disorder. For instance, patients who were more distressed and frightened during ACS showed higher levels of anxiety and depressive symptoms, one week and three months, respectively, after the cardiac event [3]. Fright and the intensity of acute pain during ACS were both associated with posttraumatic stress symptoms three months later [6,7]. We found that retrospectively assessed levels of MI-related fear of dying, helplessness, or pain were associated with posttraumatic stress symptoms after a median of 40 days following MI [8].

The aforementioned studies suggest that distress conceptualized as MI-related fear of dying, helplessness or pain might be an important clinical entity, since it is associated with negative affective risk factors for cardiovascular morbidity and mortality, including depression, anxiety, and posttraumatic stress disorder [9-11]. Virtually all descriptions of negative affect distinguish among anxiety and related constructs (e.g. fear) and depression and related constructs (e.g. helplessness) [12]. Therefore, MI-related fear of dying and helplessness could be understood as part of the negative affective spectrum being associated with poor cardiovascular prognosis in the aftermath of MI. Moreover, increasing attempts have been made to dismantle negative affective constructs in order to identify for instance the "cardiotoxic" components of depression in patients with coronary heart disease [13]. In other words, MI-related fear of dying and helplessness may seem to tap into specific qualities of negative affect, thereby having the potential to emerge as risk factors of poor cardiovascular prognosis and as specific targets for behavioral interventions in their own right. Several processes might help to explain the putative relation between MI-related distress and subsequent CVD-related events. As has been shown for other types of negative affect, these might relate to poor life style choices, low adherence with cardiac therapy, and distinct pathophysiologic processes directly harming the cardiovascular system [14].

As a first step of testing the value of MI-related distress for post-MI prognosis, we investigated the hypothesis that greater fear of dying, helplessness, or pain intensity (i.e., perceived distress during acute MI) would be associated with increased risk of future hospital readmissions due to non-fatal cardiovascular events and related interventions. We further hypothesized that MI-related distress would be associated with poor cardiovascular outcome independent of other important prognostic factors.

## Methods

### Study participants

All participants provided written informed consent to the study protocol that was approved by the ethics committee of the Canton of Bern, Switzerland, as part of the ongoing longitudinal Swiss Heart and Mind Study. The flowchart shows the recruitment of the 304 patients available for the present investigation. As previously detailed [15], between 01/2005 and 04/2007, we approached 951 consecutive patients referred to the Department of Cardiology, Inselspital, Bern University Hospital, Switzerland. Inclusion criteria were a verified acute ST-elevation or non-ST-elevation MI, living within a 90-min drive from the University Hospital, and sufficient knowledge of German. Response rate was 44.8% (426/951). Within a median of 52 days (range 12-365), participants in the present study were sent home rating scales to assess distress perceived during MI. For the follow-up investigation, patients were contacted again by mail and asked for their consent to participate in assessment of cardiovascular outcome since assessment of MI-related distress.

### Assessment of patient characteristics

Patient characteristics including sex, age, type of index MI (first-time vs. recurrent MI), left ventricular ejection fraction (LVEF) measured by ventriculography during coronary angiography, and the number of diseased coronary vessels, were abstracted from hospital charts recorded at the time of the index MI. Hypertension (yes/no) was defined by either a positive history for treatment or systolic and/or diastolic blood pressure ≥140/90 mmHg at rest. Diabetes (yes/no) was defined by a positive history that, if unclear, was verified by one-time glucose level >200 mg/dl. The status of current smoking (yes/no) was also obtained from the charts. Data on LVEF, hypertension, diabetes, and smoking status were missing in 9 (3.0%), 6 (2.0%), 14 (4.6%), and 15 (4.9%) patients, respectively. The use (yes/no) of aspirin, statins, beta blockers, and angiotensin-converting enzyme (ACE) inhibitors was noted with respective data missing in 4 (1.3%), 5 (1.6%), 9 (3.0%), and 4 (1.3%) patients, respectively.

### Assessment of distress perceived during myocardial infarction

The patients retrospectively rated three aspects of subjective perception of distress related to MI on a numeric rating ranging from 0 to 10 points [8]: a) *fear of dying: *"During my referral to the hospital, the emergency unit, or the intensive care unit, I was afraid I was dying." (0 = absolutely not true, 10 = absolutely true); b) *helplessness: *"When the doctor told me I had a heart attack, I was frightened, felt helpless, and was afraid of losing control of the situation." (0 = absolutely not true, 10 = absolutely true); c) *pain intensity*: "Please indicate how strong your pain was during the heart attack." (0 = no pain at all, 10 = intolerable pain). Cronbach's alpha for the three scales was 0.76 suggesting acceptable reliability for the measured construct of "MI-related distress".

### Assessment at follow-up

The follow-up period referred to the time interval between assessment of distress and a semi-structured telephone interview during which patients were asked whether they had been hospitalized because of a new cardiovascular event or related intervention specified as follows a priori: recurrent MI, elective and non-elective percutaneous coronary intervention with stent implantation, coronary artery bypass grafting, pacemaker implantation, cardiac arrhythmia, cardiac arrest, cerebrovascular insult, transient ischemic attack, hypertensive crisis, heart failure. A positive answer was verified by contacting the treating physician by phone. We also asked whether the patient had received mental health treatment (i.e. antidepressants, psychotherapy) and whether he or she had had non-specific chest pain after the index MI. Eleven patients confirmed the latter and were excluded from the analysis because thoracic pain might potentially affect retrospective ratings of distress.

### Statistical analysis

Data were analyzed using SPSS 15.0 statistical software package (SPSS Inc. Chicago, IL). Two-tailed level of significance was set at p < 0.05. Differences between groups were calculated using Student's *t *test, Pearson chi-square test, and Fisher's exact test where appropriate. Pearson correlation coefficients were computed to estimate the correlation between two variables. We ran three separate Cox proportional hazard models to estimate the relative risk (hazard ratio with 95% confidence interval) of a hospital readmission during follow-up because of a CVD event or CVD-related intervention (combined endpoint) as the outcome in relation to a 2-point increase of MI-related distress ratings. That is, we first assessed distress measures with the 10-point scale, then divided the score by two, and used the obtained value in analysis. Using a 0 to 10 numeric rating scale, changes of approximately 2 points or 30% to 36% represent clinically meaningful changes in pain severity [16]. For the sake of consistency, we similarly judged a change of 2 points on the 0 to 10 numeric rating scales for fear of dying and helplessness to be clinically meaningful.

**Figure 1:**
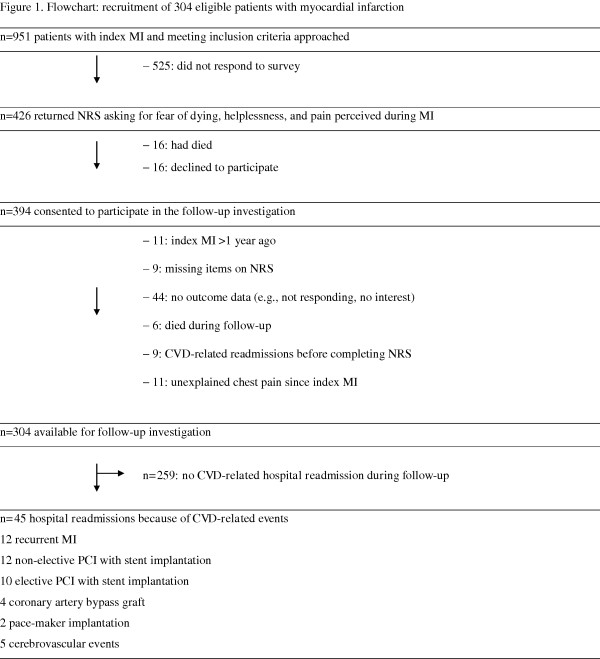
**CVD, cardiovascular disease; MI, myocardial infarction; PCI, percutaneous coronary intervention; NRS, numeric rating scale**.

To avoid overfitted and thus unstable models, the 45 outcome events (Figure 1) allowed us to force a maximum of four potentially confounding variables in addition to the respective distress measure (i.e., a maximum of five independent variables) all in one block into the equation [17]. Confounders of recurrent cardiac events in post-MI patients were defined a priori, being age [18] severity of CHD, as indexed by the number of diseased coronary vessels [19], and the major CVD risk factors hypertension [20] and smoking [21].

## Results

### Patient characteristics and cardiovascular readmissions

The median duration of follow-up after assessment of MI-related distress measures was 32 months (range 16-45) during which a CVD-related hospital readmission occurred in 45 patients (14.8%). The type of CVD events and interventions is shown in Figure 1. The characteristics of the entire sample as well as stratified by CVD-related readmission are given in Table 1. Compared to patients with no CVD-related readmission, those who experienced a cardiovascular event or related intervention were more frequently hypertensive and scored higher on all distress ratings. No group difference was seen in terms of demographic characteristics, severity of CHD, cardiac medication, and mental health treatment.

**Table 1:** Characteristics of 304 patients per cardiovascular disease readmissions

	All (n = 304)	Readmission (n = 45)	No readmission (n = 259)	p-value
Women (%)	15.5	6.7	17.0	0.115

Age (years)	60.9 ± 10.6	59.9 ± 11.1	61.0 ± 10.5	0.512

Time between MI and distress assessment (days)	74.7 ± 57.8	71.2 ± 54.5	75.3 ± 58.4	0.669

Recurrent MI (%)	9.5	11.1	9.3	0.782

1-, 2-, 3-vessel disease (%)	43.4, 32.6, 24.0	28.9, 42.2, 28.9	45.9, 30.9, 23.2	0.100

Left ventricular ejection fraction (%)	50.1 ± 10.6	50.5 ± 9.2	50.0 ± 10.9	0.794

Hypertension (%)	60.4	75.0	57.9	0.032

Diabetes (%)	11.7	9.3	12.1	0.798

Current smoker (%)	40.8	45.5	40.0	0.509

Aspirin (%)	98.3	100	98.1	1.000

Statin (%)	96.7	100	96.1	0.367

Beta blocker (%)	89.5	92.7	92.1	1.000

ACE inhibitor (%)	68.9	69.8	68.8	0.894

Antidepressants (%)	11.2	13.3	10.8	0.610

Psychotherapy (%)	7.9	4.4	8.5	0.550

Fear of dying (score)	2.71 ± 3.18	3.78 ± 3.57	2.53 ± 3.08	0.015

Helplessness (score)	2.76 ± 3.10	3.82 ± 3.54	2.58 ± 2.99	0.030

Pain (score)	6.01 ± 2.92	6.87 ± 2.91	5.86 ± 2.90	0.033

ACE, angiotensin-converting enzyme; MI, myocardial infarction

### Bivariate correlations with distress measures related to myocardial infarction

There were positive associations among all distress measures; i.e., between fear of dying and helplessness (r = 0.79, p < 0.001), fear of dying and pain (r = 0.40, p < 0.001), and helplessness and pain (r = 0.33, p < 0.001). More time elapsed since the MI correlated with greater fear of dying (r = 0.12, p = 0.032) but not significantly so with helplessness or pain. Younger age was associated with higher scores of fear of dying (r = -0.24, p < 0.001), helplessness (r = -0.23, p < 0.001), and pain (r = -0.16, p = 0.006). Smokers showed greater helplessness than non-smokers (3.35 ± 3.57 vs. 2.44 ± 2.71, p = 0.020). Patients who had received antidepressant medication indicated greater fear of dying (3.94 ± 3.63 vs. 2.56 ± 3.10, p = 0.040), greater helplessness (3.88 ± 3.42 vs. 2.62 ± 3.04, p = 0.025), and more intense pain (6.94 ± 3.11 vs. 5.90 ± 2.88, p < 0.049) during MI than those who were not prescribed antidepressants. There were no significant correlations between any distress measure and gender, hypertension, diabetes, measures of CHD severity, cardiac medications, and psychotherapy since index MI.

### MI-related distress and CVD-related hospital readmissions

As shown in Table 2, for a 2-point increase in fear of dying, helplessness, or pain, there was a respective increase of 21%, 22%, and 27% in the relative risk of a CVD-related hospital readmission without adjustment for covariates. No one distress measure turned out to be significant if entered together in one block into the equation (fear of dying: HR 1.05, 95% CI 0.79-1.39, p = 0.74; helplessness: 1.12, 95% CI 0.85-1.49, p = 0.41; pain: HR 1.18, 95% CI 0.94-1.50, p = 0.16).

**Table 2:** Unadjusted relative risk (95% CI) of distress measures for cardiovascular disease-related hospital readmissions

Distress Measure	All events (n = 304, 45 events)	Unscheduled events (n = 294, 35 events)
Fear of dying	1.21 (1.03-1.43)	1.26 (1.05-1.51)
	p = 0.020	p = 0.012
Helplessness	1.22 (1.04-1.44)	1.26 (1.05-1.52)
	p = 0.017	p = 0.013
Pain	1.27 (1.02-1.58)	1.30 (1.01-1.66)
	p = 0.033	p = 0.042

Relative risks are expressed for a 2-point increase on numeric rating scales for distress measures. Unscheduled events do not include elective stent implantation that occurred during follow-up.

It is possible that at the time of distress assessment, the 10 patients who underwent elective stenting during follow-up already knew about a planned readmission such that they might differ in their distress ratings from the 35 patients who experienced an unscheduled CVD-related event. Therefore, we conducted a sensitivity analysis excluding patients having undergone elective stent implantation; this analysis showed an increase in the relative risk for an unscheduled CVD-related hospital readmission of 26%, 26%, and 30%, respectively, for a 2-point increase in fear of dying, helplessness, or pain (Table 2).

Table 3 shows the multivariate-adjusted hazard models that included 286 patients of whom 43 experienced a CVD-related hospital readmission during follow-up. Compared to the results from the unadjusted analysis (Table 2), the effect size of the relationship between a 2-point increase in any distress measure and the relative risk of a CVD-related event was maintained or increased even slightly when taking into account age, the number of diseased coronary vessels, hypertension, and smoking. Hypertension, but not age, the number of diseased coronary vessels, and smoking emerged as a significant predictor of outcome in all of the three multivariate models.

**Table 3:** Multivariate-adjusted relative risk (95% CI) of distress measures for cardiovascular disease-related hospital readmissions

Entered variables	Fear of dying	Helplessness	Pain
Fear of dying	1.24 (1.04-1.46)	--	--
	p = 0.015		
Helplessness	--	1.26 (1.06-1.50)	--
		p = 0.010	
Pain	--	--	1.26 (1.01-1.57)
			p = 0.042
Age	1.01 (0.87-1.19)	1.01 (0.87-1.18)	0.99 (0.85-1.15)
	p = 0.86	p = 0.87	p = 0.92
1-, 2-, 3-vessel disease	1.27 (0.88-1.84)	1.27 (0.88-1.83)	1.26 (0.87-1.83)
	p = 0.20	p = 0.21	p = 0.22
Hypertension	2.10 (1.02-4.36)	2.22 (1.07-4.60)	2.13 (1.03-4.39)
	p = 0.046	p = 0.033	p = 0.040
Smoking	1.24 (0.66-2.36)	1.22 (0.64-2.32)	1.20 (0.63-2.30)
	p = 0.50	p = 0.56	p = 0.58

Model statistics	χ^2 ^= 14.01, df = 5,	χ^2 ^= 14.64, df = 5,	χ^2 ^= 12.03, df = 5,
	p = 0.016	p = 0.012	p = 0.034

All covariates were entered in one block together with the respective distress measure. Relative risks are expressed for a 2-point increase on numeric rating scales for distress measures and for a 5-year increase for age (i.e., age values were divided by 5 before entering the equation). Because of missing data for hypertension and smoking status, all models included 286 patients and 43 cardiovascular disease-related events.

## Discussion

We investigated the association between retrospectively rated MI-related fear of dying, helplessness, or pain intensity and non-fatal CVD outcome. These measures showed acceptable reliability for a construct of "MI-related distress" and they are also shown to be clinically important because of their predictive value for poor psychological adjustment during recovery from MI [3,6-8]. We found that MI-related distress was associated with an increased risk of hospital readmissions due to cardiovascular events and related interventions during a mean follow-up of almost three years. This association was independent of potentially important prognostic factors, namely age, coronary heart disease severity, hypertension and smoking: of these, hypertension alone emerged as a significant predictor of event risk. The relations between MI-related distress measures and CVD event risk seems relevant, as a change of 2 points (or between 30% and 36%) in pain intensity on numeric rating scales ranging from 0 to 10 is considered to be clinically meaningful [16]. In our patients a 33% increase in distress severity would mean an increase in distress scores from 6 to 8 corresponding to a 1.2- to 1.4-fold increased risk of CVD-related hospital readmissions.

Our study is on the one hand to be understood as a first attempt of tracking down the prognosis of post-MI patients who perceive their MI as stressful. On the other, it suggests that MI-related distress does not only predict psychological adjustment post-MI, as was previously shown [3,6-8], but also CVD outcome. In other words, the focus of our study was to investigate the possibly direct association between distress and poor CVD outcome in post-MI patients by taking demographic factors, CHD severity, and major CVD risk factors into account. However, because psychological adjustment post-MI was variously predicted by distress measures, including fear of dying, helplessness, or pain intensity [6-9], the extent to which distress is associated with poor CVD prognosis independent from its psychological sequel remains unresolved.

Patients reporting greater levels of all distress measures received more frequently antidepressant medication during follow-up. However, we do not know the type of antidepressants our patients received. For instance, particularly selective serotonin reuptake inhibitors seem to improve CVD outcome [22]. Further elucidation of the likely complex psychological pathways leading from MI-related distress to poor CVD prognosis is warranted, because these might provide cues for tailored behavioral interventions. For instance, patients might profit from reassurance and provided safety during MI [3] and later on from more trauma-focused cognitive behavioral therapy [23]. Our observation that distress measures correlated inversely with age might indicate that younger patients are in particular need of psychological support during MI.

In addition to psychological maladjustment, other explanations for the association between greater MI-related distress and an increased future risk of CVD events might relate to an unhealthy life style, poor compliance with cardiac therapy, and psychophysiologic alterations [14]. In our study, smokers showed greater helplessness than non-smokers. In another study, good medical recovery from MI was associated with positive life orientation, which in turn correlated inversely with helplessness [24]. Psychological stress is also associated with an unhealthy diet, physical inactivity, and sleep disturbances, all of which may impact cardiovascular health [14], but were not available in our study. Particularly distress and fear during ACS were shown to be lower in regular exercisers than in patients who exercised less frequently [3]. Depression and PTSD compromise prescribed intake of cardiac medication [25,26], thereby suggesting another pathway leading from distress via psychological maladjustment and poor adherence to increased CVD risk. Future studies may also want to investigate the physiologic correlates of distress during MI to investigate their trajectories and predictive value for CVD-related events. For instance, there is some evidence that elevated heart rate and lowered cortisol in the immediate aftermath of a psychological trauma predict the development of posttraumatic stress symptoms [27,28]. However, it is unknown how this might affect cardiovascular biology in the longer run.

We observed different results when entering all distress measures simultaneously into the survival analysis, namely that distress was no longer associated with outcomes. Because the inter-correlation among the three distress measures was substantial, one statistical explanation could be that their separate effects partialled out each other. Another explanation could be that none of the distress measure components was associated with outcomes above and beyond one another suggesting that they might be equally important in predicting cardiac outcome individually. In other words, as the three distress measures might substitute for each other, it might seem unnecessary to measure all of them in solitude. However, future studies may want to test how distress measures as proposed here and possibly others might best be integrated into a unifying measure of distress to reliably predict cardiac prognosis after MI.

We mention several limitations of our study. Although comparable with our studies in this field, the response rate of 44.8% of the originally approached 951 patients was rather low, and, as previously reported, women responded less than men [9]. This might limit the generalizibility of our results to the general post-MI population and particularly women patients. We assessed MI-related distress retrospectively bearing the risk of biased reporting because of concomitant negative affect. We did not assess negative affect like depression and anxiety to control our results for this possibility. However, another study found only borderline significance between a negative affect scale and distress (including fear of dying) during ACS [3]. Patients varied considerably in time since index MI which might have variably affected distress measurements; for instance, fear of dying seemed to be greater with more time elapsed since the index MI. We excluded patients who had reported unexplained chest pain since index MI but we did not have data available on symptoms such as thoracic pain the patients and their physicians might have attributed to the heart. Such symptom attributions might potentially affect retrospective reports of MI-related distress. There might be events, which have happened during the time since index MI which may contribute to the retrospective evaluation of distress (e.g., familial difficulties, death or other illnesses in the family, economical problems) for which we could not control our analysis. The number of outcome events limited the adjustment of hazard models for additional potentially important confounding variables like sex and diabetes.

## Conclusions

The findings from this study suggest that retrospectively assessed MI-related distress in the form of fear of dying, helplessness, or pain intensity is associated with an increased risk of future non-fatal cardiovascular events and related interventions. Numeric rating scales to assess symptom severity (incl. pain) are widely used in clinical settings. Particularly, the numeric rating scales applied in this study to measure distress are easy administrable even in a busy clinical setting and thus of potential clinical applicability in screening post-MI patients at risk of poor cardiovascular outcome. The association between MI-related distress and poor cardiac outcome was independent of other important prognostic factors. The downstream psychopathology and behavior as well as the underlying physiology of this association remain to be elucidated.

## Competing interests

The authors declare that they have no competing interests.

## Authors' contributions

All authors participated in the design of the study, helped to draft the manuscript and read and approved the final manuscript. RvK performed statistical analysis and wrote the first draft of the manuscript. RH performed all the telephone interviews. RH and JPS collected all the additional data reported in this manuscript. RvK, SB and HS critically supervised data acquirement and made important intellectual contribution to the interpretation of the data.

## Pre-publication history

The pre-publication history for this paper can be accessed here:

http://www.biomedcentral.com/1471-244X/11/98/prepub
